# Anti-PD-1 sintilimab-induced bilateral optic neuropathy in non-small cell lung cancer: A case report and literature review

**DOI:** 10.3389/fonc.2022.931074

**Published:** 2022-08-09

**Authors:** Jian Wang, Xiaoyue Xiao, Xiaorong Dong, Gang Wu, Xinghua Wang, Ruiguang Zhang

**Affiliations:** ^1^ Cancer Center, Union Hospital, Tongji Medical College, Huazhong University of Science and Technology, Wuhan, China; ^2^ Institute of Radiation Oncology, Union Hospital, Tongji Medical College, Huazhong University of Science and Technology, Wuhan, China; ^3^ Department of Cardiovascular Surgery, Union Hospital, Tongji Medical College, Huazhong University of Science and Technology, Wuhan, China; ^4^ Department of Ophthalmology, Union Hospital, Tongji Medical College, Huazhong University of Science and Technology, Wuhan, China

**Keywords:** non-small cell lung cancer, immune-related adverse events, optic neuropathy, PD-1, sintilimab

## Abstract

Anti-PD-1/PD-L1 immunotherapy reactivates T-cell activity to boost the antitumor effect and may trigger autoimmune toxicity in various organ systems involving eyeball and periocular structures at the same time. The rarity of ocular immune-related adverse events should not prevent us from paying attention to this issue because of the bad consequences of visual impairment. This is the first case report of anti-PD-1 sintilimab-induced bilateral optic neuropathy in a 76-year-old man with squamous non-small cell lung cancer (NSCLC). The patient presented with sudden vision blurring without pain in both eyes after three therapeutic cycles of sintilimab plus chemotherapy. Based on the ophthalmic examination, laboratory, and radiological results, our patient was diagnosed with optic neuropathy complication secondary to anti-PD-1 sintilimab treatment. Consequently, sintilimab was held and systemic steroids were administered. The follow-up review showed that the vision recovered and the size of the primary tumor continued to decrease with the response assessment as the partial response. In conclusion, this case report suggested that patients with NSCLC undergoing anti-PD-1/PD-L1 therapy should be closely monitored for ophthalmic assessment and alert to the occurrence of sintilimab-induced optic neuropathy.

## Introduction

Non-small cell lung cancer (NSCLC) is one of the most prevalent and aggressive malignant tumors, which causes the most cancer-related death worldwide (1). Next to lung adenocarcinoma, lung squamous cell carcinoma (LUSC) is the second most common histologic subtype of NSCLC, accounting for about 25%–30% of cases in NSCLC (2, 3). Surgical resection, radiotherapy, chemotherapy, and immunotherapy are the mainstays in the treatment of LUSC. Currently, immuno-oncology agents, such as antibodies targeting programmed death 1 (PD-1) protein or its ligand PD-L1, have achieved great success and revolutionized cancer therapy (4).

The PD-1/PD-L1 blocking antibodies disturb the binding of PD-L1 to PD-1 on T cells, thus reversing T-cell exhaustion and boosting antitumor immunity (5). An indirect comparison analysis indicated that the anti-PD-1 antibody pembrolizumab might have superior efficacy compared to anti-PD-L1 antibody atezolizumab in combination with chemotherapy for LUSC especially in PD-L1 low/negative patients (6). Recently, the immunotherapeutic anti-PD-1 antibodies have shown advantageous and beneficial anti-tumor effects in LUSC. In patients with treatment-naive metastatic LUSC, the addition of pembrolizumab to chemotherapy with carboplatin plus paclitaxel or nab-paclitaxel provided a significant improvement in overall survival (OS) and progression-free survival (PFS) versus chemotherapy alone (KEYNOTE-407) (7). Patients treated with Nivolumab were observed with significantly better OS, response rate, and PFS than with docetaxel in patients with advanced previously treated LUSC (CheckMate 017/057) (8, 9). In an international, randomized phase 3 trial (CheckMate 9LA), first-line nivolumab plus ipilimumab combined with two cycles of chemotherapy resulted in significantly longer OS than chemotherapy alone in patients with squamous NSCLC and non-squamous NSCLC (10). The phase 3 randomized clinical trial (RATIONALE 307) demonstrated that Tislelizumab plus chemotherapy resulted in significant improvement of PFS and objective response rate (ORR) compared with chemotherapy alone with a manageable safety/tolerability profile (11). The phase 3 randomized trial (ORIENT-12) showed that sintilimab plus gemcitabine and platinum offered a meaningful improvement in PFS than the placebo-chemotherapy group (12).

With the widespread use of immunotherapy in cancer therapy globally, immunotherapeutic toxicities have become a very critical topic that requires immediate diagnosis and treatment (13). Meanwhile, some rare but life-threatening anti-PD-1 antibody-related side effects were recognized and reported in the management of NSCLC (14). Ophthalmic immune-related adverse events (irAEs) have been reported in less than 5% of patients treated with anti-PD-1 antibody-containing regimens (15). However, untimely recognition and inappropriate treatments can be sight-threatening and finally impair the quality of life (16).

A comprehensive review reported that ophthalmoplegia (40.51%), uveitis (20.25%), dry eye (17.72%), retinopathy (5.06%), conjunctivitis (5.06%), and optic neuritis (3.80%) were the most common immune checkpoint inhibitor (ICI)-related ocular adverse events in lung cancer (17). A recent report showed that pembrolizumab might lead to inflammatory optic neuritis, which could reverse by the cessation of ICI and the use of corticosteroids (18). Sintilimab, as one of the most successful anti-PD-1 inhibitors, has been moved to the forefront of cancer treatment and is increasingly being administered as part of therapy for LUSC (12, 19). Here, we present the first case of sintilimab-induced bilateral optic neuropathy following three cycles of sintilimab and chemotherapy in NSCLC.

## Case presentation

**Figure 1:**
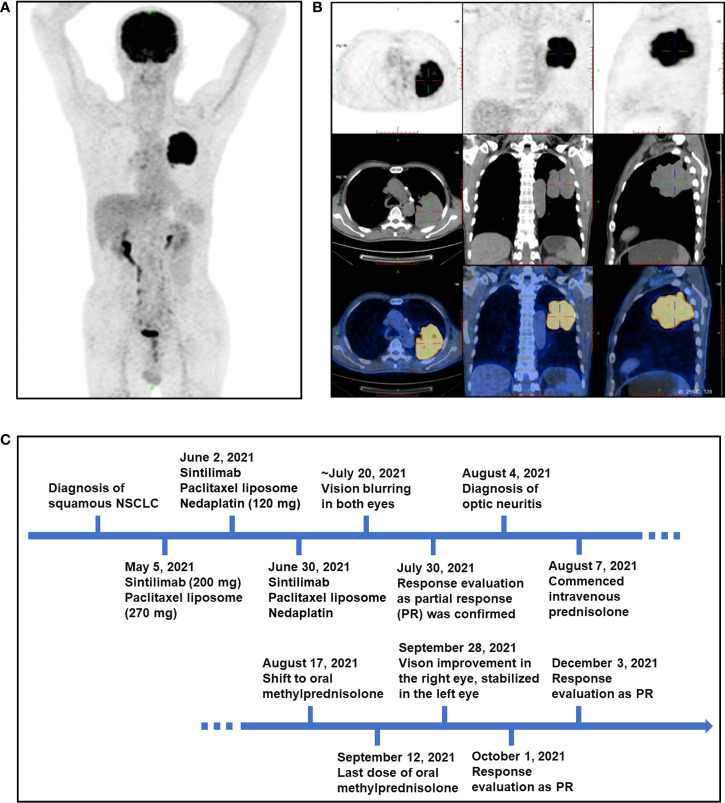
PET-CT scan of the patient at baseline and an outlined timeline of his treatment and adverse events. **(A, B)** Representative images of PET-CT scan of the patient at baseline on 5 May 2021. **(C)** The schematic timeline for the patient, from diagnosis to resolution of optic neuropathy (not to scale).

The patient was a 72-year-old man diagnosed with stage IIIB cT4N2-3M0 squamous subtype NSCLC in March 2021. As to physical examination on presentation to the hospital, the patient’s body temperature was 36.2°C, blood pressure was 109/68 mmHg, pulse was 89 beats/min, heart rate was 89 beats/min, respiratory rate was 18 breaths/min, and body mass index (BMI) was 20.5 kg/m^2^. The Karnofsky score was 90. His past medical issues included appendectomy (9 years ago), cataract phacoemulsification and intraocular lens implantation in both eyes (5 years ago), and type 2 diabetes mellitus (diagnosed in March 2021, treated with linagliptin, 5 mg po, qd and acarbose 50 mg, po, tid). He had no relevant previous comorbidities, and no previous history of liver disease, hepatitis, alcohol abuse, or smoking. Meanwhile, there was no medical history of autoimmune diseases and endocrinopathies. The positron emission tomography-computed tomography (PET-CT) scan showed that irregular soft tissue masses (approximately 8.7 × 6.0 × 7.7 cm) in the dorsal segment of the left upper lobe and lower lobe of the left lung are lobulated, growing across the interlobular fissures, local bronchial occlusion, and abnormally increased standard uptake value (SUV) activity, which is consistent with lung cancer manifestations (**Figures 1A, B**). Some of the left hilar lymph nodes, mediastinal lymph nodes and right hilar lymph nodes displayed increased SUV activity. Metastatic lesions in the other organs or systems were not observed. The bronchoscopic needle biopsy was performed and immunohistochemistry staining showed CK5/6 (+), CK7 (-), PCK (+), Ki67 (LI: about 20%), Napsin A (-), P40 (+), P63 (+), TTF-1 (-), and ALK (D5F3) (focal +), which supported a diagnosis of squamous NSCLC.

**Figure 2:**
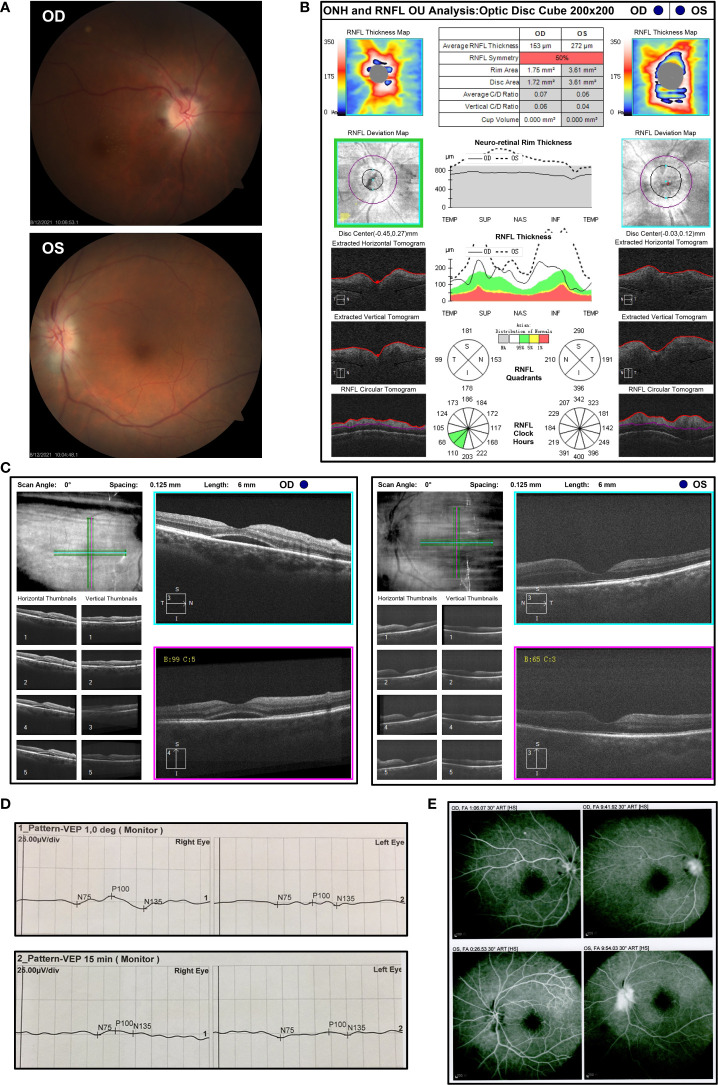
The ophthalmic examination of the patients with bilateral optic neuropathy after three cycles of sintilimab-containing therapy **(A)** Fundus photography displayed obvious optic disc edema in both eyes with macular edema in his right eye and parapapillary linear hemorrhage in his left eye after the third cycle of sintilimab plus chemotherapy on 12 August 2021. OD, oculus dexter; OS, oculus sinister. **(B)** Measurements of the thickness of retinal nerve fiber layer (RNFL) by optical coherence tomography (OCT) on 5 August 2021. **(C)** Macular OCT showed subretinal fluid in the right eye on 5 August 2021. **(D)** Visual evoked potential (VEP) was measured on 6 August 2021 and showed prolonged P100 latency and decreased P100 amplitude at 60 and 15 arcmin in both eyes. **(E)** Fundus fluorescein angiography (FFA) indicated optic disc fluorescence leakage at the late stage and low fluorescence perfusion at the early stage in both eyes on 12 August 2021.

Treatment with sintilimab (200 mg) plus paclitaxel liposome (270 mg) was initiated on 5 May 2021. He then received two cycles of therapy including sintilimab plus paclitaxel liposome (270 mg) and nedaplatin (120 mg) on 2 June 2021 and 30 June 2021. Three weeks after the third infusion of sintilimab, he presented with sudden vision blurring in his both eyes without redness, eye pain, swelling, or other discomforts. No special treatment was performed outside. Approximately 2 weeks after he first developed vision blurring, the patient was referred to the Department of Ophthalmology (**Figure 1C**). On ophthalmic examination, the best corrected visual acuity (BCVA) was 20/300 in his right eye and finger count (eye front) in his left eye. The intraocular pressure was 15 mmHg in both eyes. The eye position was normal without proptosis, and the eye movement was normal in all directions. In addition, no obvious redness or swelling was observed in the eyelid and conjunctiva. By the slit lamp examination, the cornea was transparent and the anterior chamber was clear without Tyndall in both eyes. The pupils were round with a diameter of about 3 mm, and sensitive to light reflection. The intraocular lenses were in the right position. Fundus photography showed obvious optic disc edema in both eyes, accompanied by macular edema in his right eye and parapapillary linear hemorrhage in his left eye (**Figure 2A**). By optical coherence tomography (OCT), the average retinal nerve fiber layer (RNFL) thickness was 153 μm and 275 μm in right and left eye, respectively (**Figure 2B**). Also, subretinal fluid was noticed in the macular region of the right eye (**Figure 2C**). Visual evoked potential (VEP) showed prolonged P100 latency and decreased P100 amplitude at 60 and 15 arcmin in both eyes (**Figure 2D**). However, the visual field examination could not be performed because of poor vision. Fundus fluorescein angiography (FFA) showed optic disc fluorescence leakage at the late stage and low fluorescence perfusion at the early stage in both eyes, which suggested anterior ischemic optic neuropathy (**Figure 2E**). Magnetic resonance imaging (MRI) of the brain with gadolinium enhancement was negative for metastatic lesions, recent infarcts, or enhancement in the optic nerve (**Figure S1**) on 31 July 2021. No orbital abnormality was detected, and the remaining findings of the neurological system and other systems were unremarkable. The patient refused to undergo a lumbar puncture and hence cerebrospinal fluid analysis is lacking. Laboratory evaluations including general biochemistry and serological tests were basically normal, and the results are shown in **Tables S1–S5**. The C-reactive protein was elevated to the level at 22.8 mg/L.

**Figure 3:**
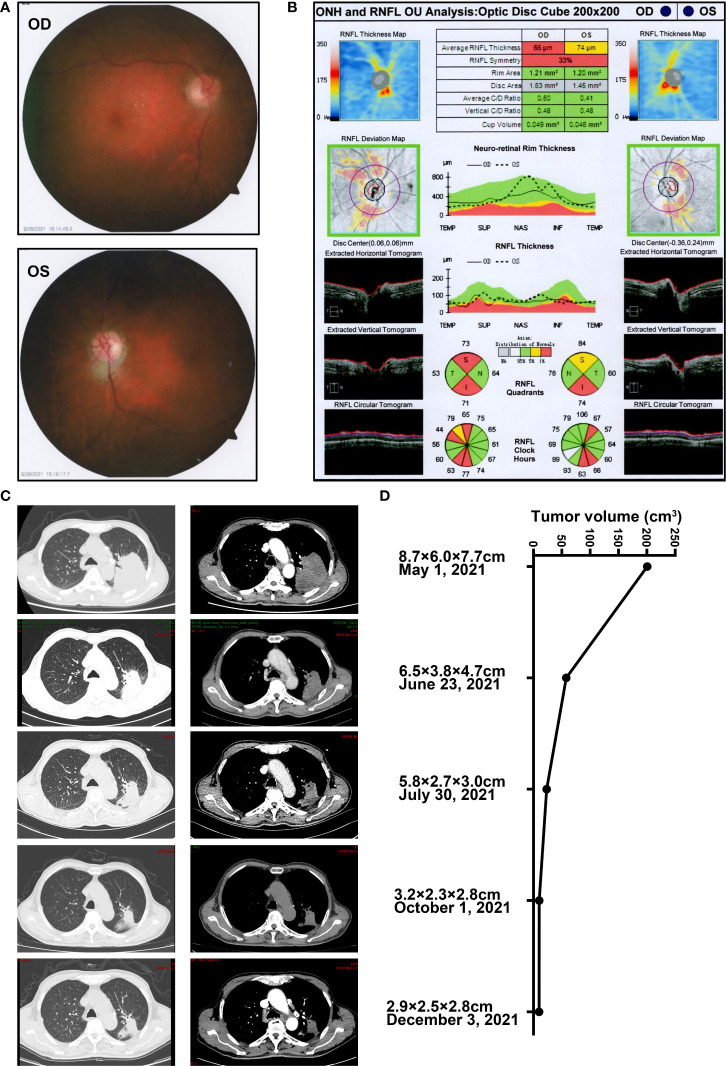
The ophthalmic examination of the patients post steroids against sintilimab-induced optic neuropathy and representative CT images in the follow-up **(A)** Fundus photography post steroids against optic neuropathy secondary to sintilimab therapy on 28 September 2021. **(B)** OCT examination of the optic disc post steroids against sintilimab-induced optic neuropathy on 28 September 2021. **(C)** Representative CT images and **(D)** the estimated tumor volume during the treatment of the case in the follow-up from 1 May 2021, to 3 December 2021. The horizontal axis represents changes in tumor volume. The vertical axis depicted the time of CT scan and the estimated tumor longitudinal diameter (X) and the greatest transverse diameter (Y) in the axial plane and the maximum thickness in head-to-foot direction (Z). The tumor volume was calculated by using the formula (X*Y*Z)/2.

Generally, anti-PD-1 therapy activates the T-lymphocyte response against tumor cells while these unrestrained T cells can also aberrantly target normal tissue and trigger a range of adverse inflammatory events (20, 21), including ophthalmic toxicity (22). Considering that immunotherapy might induce ischemia by various mechanisms and optic disc edema was severe, the presenting bilateral optic neuropathy (23) was considered as a highly probable sintilimab-related adverse event, and discontinuation of the drug was decided (21, 24). Consequently, systemic steroids and neurotrophic therapy were recommended. The regimen could consider high-dose methylprednisolone pulse treatment (methylprednisolone 10–20 mg/kg/day intravenously and reduced to 5–10 mg/kg/day for 3 days), then 1 mg/kg/day for 3 days, followed by tapering oral methylprednisolone slowly over 4–6 weeks (25). Considering the patient’s advanced age and the history of tumor, he preferred commencing intravenous prednisolone 60 mg (1 mg/kg/day) daily for five consecutive days and reducing it to 30 mg (0.5 mg/kg/day), then shifting to oral methylprednisolone (started at the dose 0.4 mg/kg/day) with a long tapering over 4 weeks after discharge. About 2 months after the onset of symptoms and while steroid treatment was discontinued for 2 weeks, BCVA recovered to 20/50 in his right eye, and BCVA of the left eye stabilized. Fundus photography showed a slightly pale optic disc with a clear boundary (**Figure 3A**). OCT suggested that the thickness of RNFL in both eyes became thinner than the normal range, exactly 65 μm in his right eye and 74 μm in his left eye (**Figure 3B**). The CT imaging post-sintilimab plus chemotherapy demonstrated a marked reduction of tumor size, consistent with a partial response (PR) to treatment (**Figures 3C, D**). Results of the tumor markers in serum displayed that the level of squamous cell carcinoma antigen (SCC) reduced to the normal level and the levels of cytokeratin-19-fragment CYFRA21-1, carcinoembryonic antigen (CEA), and carbohydrate antigen 125 (CA125) were also significantly decreased, while CA19-9 and CA15-3 remained within the reference range (**Table S6**). At 5 months’ follow-up, the patient is clinically well and remained off rechallenge with sintilimab or other systemic antitumor treatment. Moreover, sequential chest CT scans demonstrated an ongoing trend to tumor shrinkage with the response evaluation as the partial response (PR) even after the discontinuation of systemic antitumor therapy.

## Discussion

In the era of anticancer immunotherapies, irAEs secondary to anti-PD-1 antibodies have been growingly reported with the broad administration of ICIs (13, 26). The immunoregulatory molecule PD-1 is expressed on the cell surface of lymphocytes and inactivates T cells by binding to its PD-L1 ligand on tumor cells, thus terminating immune response after antigen activation (5). Endogenous PD-1/PD-L1 signaling is key to immunosurveillance as well as an important target for cancer immunotherapy (4). Sintilimab, similar to other anti-PD-1 antibodies, blocks the PD-1/PD-L1 signaling in the tumor microenvironment and subsequently reactivates T cells to amplify the antitumor immunity (4, 5). Sometimes this uncontrolled release of T-cell inhibition can trigger a course of immune events, thus causing the irAEs with sintilimab (19, 26). Although sintilimab has shown a potent efficacy in the treatment of tumors with manageable toxicity profiles, the side effects of sintilimab, including pneumonitis, anemia, diarrhea, colitis, hepatitis, nephritis, endocrinologic, hematologic, dermatologic, and other irAEs are inescapable (13, 19, 26). As the usage of sintilimab increases, some rare side effects have been documented, among them being neuro-ophthalmic complications of ICIs (12, 19, 27).

In general, ICIs associated toxicities affect more than half of the patients treated with anti-PD-1/PD-L1 therapy in NSCLC (14, 19, 26). The relatively rare ophthalmic irAEs have been only reported in less than 5% of patients treated with anti-PD-1 antibody-containing regimens (15). The optic neuritis was the fifth most common ICI-related ocular adverse event in lung cancer, and most of the patients with ocular irAEs other than ophthalmoplegia could be relieved after drug withdrawal and glucocorticoid treatment (17, 27). Optic neuritis is one of the ophthalmic emergencies and may lead to permanent vision damage if left inappropriately treated (16). There are some case reports of bilateral or unilateral optic neuropathy associated with anti-PD-1/PD-L1 therapy in NSCLC. However, to the best of our knowledge, we convey the first case of sintilimab-induced bilateral optic neuropathy following three cycles of sintilimab injection in a patient with squamous NSCLC. The use of sintilimab was considered as the cause of optic neuropathy, for there were no other potential factors such as personal or family history of autoimmune diseases, viral infection, ocular metastasis or use of drugs that induce optic neuropathy, and only sintilimab could be identified (28, 29).

A retrospective cohort of 11 patients (three of which were NSCLC) reported that the common three classes of ICIs (anti- CTLA4, anti-PD-1, and anti-PD-L1) have the potential to induce optic neuritis (21). The immunotherapy cycles at the onset of ocular symptoms ranged from 2 to 95 cycles with a median of four drug cycles before ICI-associated optic neuritis. The ICI-associated optic neuritis has unique clinical features of bilateral with the painless visual decline in the context of intact color vision, which is not entirely consistent with “classical” optic neuritis. The potential consequences of ICI-associated inflammation and ischemia may play a role in the pathogenesis and systemic steroids could relieve optic neuropathy. In our case, the total time from the initiation of sintilimab therapy to the onset of the ocular symptoms was about 10 weeks with three cycles of sintilimab treatment finished.

A case report showed that a 64-year-old man with stage IV NSCLC received the third-line anti-PD-L1 antibody (atezolizumab) treatment for about 12 months, which decreased tumor burden. The anti-PD-L1 therapy was withdrawn because of the general fatigue, anorexia, diarrhea, and bilateral upper limb pain, then the visual loss was noted in the left eye without eye movement pain 1 week later (30). Further testing indicated that the patient was diagnosed with optic neuritis and hypopituitarism secondary to atezolizumab and steroid pulse therapy was effective for optic neuritis. A 76-year-old Caucasian man with metastatic NSCLC presented with vision loss in his left eye after receiving three cycles of pembrolizumab (the total time from the initiation of pembrolizumab therapy to the onset of the ocular symptoms was 72 days) (18). On presentation, BCVA was 20/30 in the right eye and 20/200 in the left eye. Fundoscopy revealed optic nerve edema in the left eye. Visual fields examination in the right eye revealed an enlarged blind spot and an extended defect in the inferior nasal quadrant. In the left eye, a partial superior arcuate defect and an extended defect in the inferior hemisphere were observed. The mean deviation was −12.15 dB in the right eye and −13.70 dB in the left eye. Pembrolizumab was withheld and systemic corticosteroids were administered for a total of 9 weeks resulting in the recovery of the left isolated optic neuritis. There were some anti-PD-1 nivolumab-associated optic neuritis cases reported in other solid cancer, including cutaneous melanoma (20) and glioblastoma multiforme (31).

Moreover, some previous studies suggested that irAEs might be related to stronger antitumor immunity and better tumor control, which could serve as a predictive surrogate marker of clinical benefit (32, 33). A real-world analysis and a retrospective analysis demonstrated that irAE occurrence was significantly associated with response to anti-PD-1 therapy and improved survival in PD-1-treated melanoma (34, 35). Patients developing irAEs had longer survival, especially when affecting the skin in advanced NSCLC patients receiving PD-(L)1 inhibitors (36). A multicenter retrospective study showed that multisystem irAEs were associated with related with improved survival in stage III/IV NSCLC patients treated with anti-PD-(L)1 ICIs alone or in combination with another anticancer agent (37). A large sample size analysis of NSCLC patients receiving anti-PD-1 immunotherapy indicated that irAEs of any grade might be an independent predictor of higher ORR, longer PFS, and longer OS (38). In the follow-up of our case after sintilimab cessation, the repeat chest CT scan showed stable disease with a trend of tumor shrinkage. Although there is much evidence showing the relationship between irAEs and treatment efficacy, more solid data were warranted to support this conclusion. From this case report, we learned that ophthalmic assessment is valuable in patients with NSCLC undergoing anti-PD-1/PD-L1 therapy and is helpful to identify the sintilimab-induced optic neuropathy early. The items of normal ophthalmic evaluation should include a history, physical examination, vision, visual field, fundus photography and macular OCT. The follow-up examination is recommended by the first week and the 1st/3rd/6th/12th month in the first year after the initial immunotherapy, and then annually. The testing should be done immediately if there is any abnormality in the eyes such as vision loss.

## Conclusion

This is the first case report that conveyed anti-PD-1 sintilimab-induced bilateral optic neuropathy characterized by painless vision decline in a patient with squamous NSCLC receiving three cycles of sintilimab injection. Visual function improved with sintilimab cessation and systemic steroids while the primary tumor showed an ongoing trend to reduce with the response assessment as PR (39).

## Data availability statement

The original contributions presented in the study are included in the article/**Supplementary Material**. Further inquiries can be directed to the corresponding authors.

## Ethics statement

Ethical review and approval was not required for the study on human participants in accordance with the local legislation and institutional requirements. The patient provided their written informed consent to participate in this study and consent for publication of this case report and any accompanying images.

## Author contributions

JW, XW, and RZ collected the clinical information and wrote the manuscript. XX, XD, and GW contributed to revising the manuscript critically for important intellectual content. XW and RZ coordinated the patient’s care and provided the clinical data. RZ supervised this study. All authors contributed to the article and approved the submitted version.

## Acknowledgments

We would like to thank the patient in this study and all the clinicians for providing care to the patient.

## Conflict of interest

The authors declare that the research was conducted in the absence of any commercial or financial relationships that could be construed as a potential conflict of interest.

## Publisher’s note

All claims expressed in this article are solely those of the authors and do not necessarily represent those of their affiliated organizations, or those of the publisher, the editors and the reviewers. Any product that may be evaluated in this article, or claim that may be made by its manufacturer, is not guaranteed or endorsed by the publisher.
