# Supplementary material for: Anti-PD-1 sintilimab-induced bilateral optic neuropathy in non-small cell lung cancer: A case report and literature review
Source: Front Oncol. 2022 Aug 9;12:931074. doi: 10.3389/fonc.2022.931074 (PMC9396260; doi:10.3389/fonc.2022.931074)
Supplement: Supplementary file 1 [file DataSheet_1.docx]

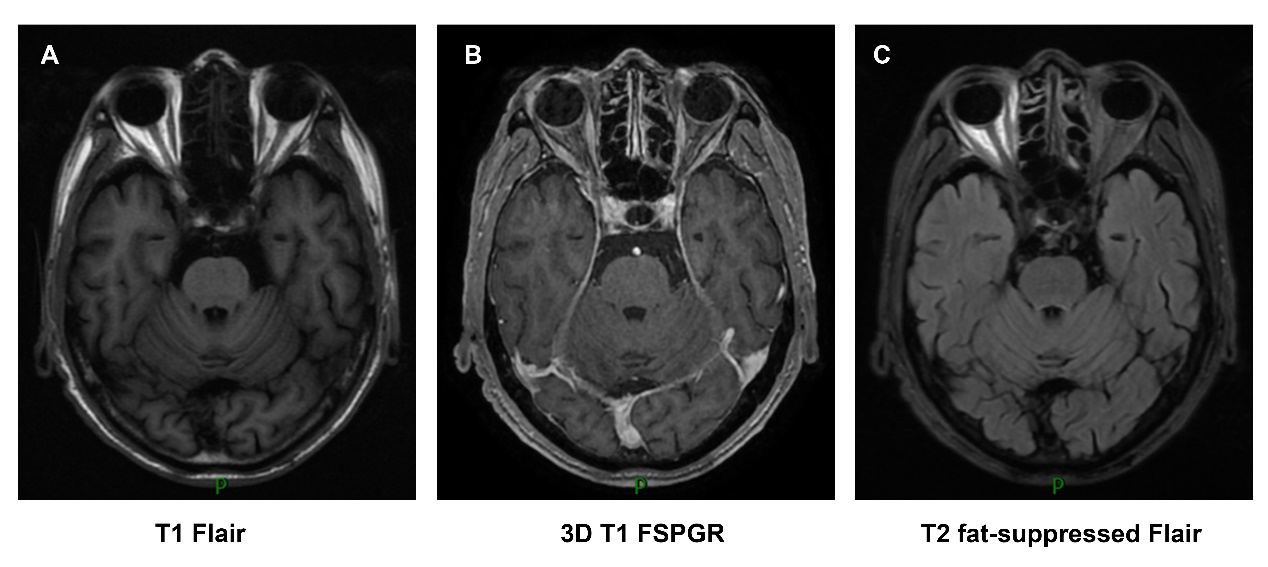


Figure S1. The gadolinium-enhanced magnetic resonance imaging (MRI) of head in the axial view.

(A) On T1 Flair sequence. (B) On 3D T1-weighted sequence. (C) On fat-suppressed T2 Flair sequence.

**Table S1 General biochemistry analysis in serum on August 5, 2021**

| **Tests** | **Results (normal values in parentheses)** |
| --- | --- |
| Total bilirubin | 6.0 μmol/L (5.1-19.0) |
| Direct bilirubin | 2.9 μmol/L (1.7-6.8) |
| Alanine aminotransferase (ALT) | 53 U/L (5-40) |
| Aspartate aminotransferase (AST) | 55 U/L (8-40) |
| Alkaline phosphatase (ALP) | 88 U/L (40-150) |
| Leucine aminopeptidase (LAP) | 33 U/L (12-37) |
| 5'-Nucleotidase | 9 U/L (0-10) |
| γ-Glutamyltransferase (GGT) | 54 U/L (11-50) |
| Total protein | 66.0 g/L (64-83) |
| Albumin | 35.6 g/L (35-55) |
| Globulin | 30.4 g/L (20-30) |
| Albumin/globulin ratio | 1.2 (1.5-2.5) |
| Prealbumin | 0.190 g/L (0.20-0.40) |
| Total bile acid (TBA) | 4.4 μmol/L (0-10.0) |
| Blood urea nitrogen (BUN) | 5.01 mmol/L (2.9-8.2) |
| Enzymatic creatinine | 104.5 μmol/L (44.0-133.0) |
| Uric acid | 459.7 μmol/L (208-428) |
| Cystatin C | 1.35 mg/L (0.63-1.25) |
| Fasting blood glucose level | 6.7 mmol/L (3.9-6.1) |
| Total cholesterol | 2.82 mmol/L (<5.2) |
| Triglycerides | 1.03 mmol/L (<1.7) |
| High-density lipoprotein (HDL) cholesterol | 0.81 mmol/L (1.16-1.42) |
| Low-density lipoprotein (LDL) cholesterol | 1.29 mmol/L (2.7-3.1) |
| Small dense low-density lipoprotein (sdLDL) cholesterol | 0.330 mmol/L (0.234-1.378) |
| Creatine kinase | 52 U/L (38-174) |
| Lactate dehydrogenase (LDH) | 277 U/L (109-245) |
| Alpha-hydroxybutyrate dehydrogenase (α-HBDH) | 199 U/L (72-182) |
| Sodium | 141.5 mmol/L (136-145) |
| Potassium | 4.40 mmol/L (3.5-5.2) |
| Chlorine | 102.3 mmol/L (96-108) |
| Homocysteine | 14.3 μmol/L (<20) |
| Calcium | 2.25 mmol/L (2.03-2.54) |
| Total carbon dioxide | 27.4 mmol/L (21.0-30.0) |
| Phosphorus | 1.11 mmol/L (0.96-1.62) |
| Magnesium | 1.00 mmol/L (0.70-1.1) |
| Anion gap | 12 mmol/L (8-16) |
| Osmotic pressure | 303.5 mOsm/L (280-310) |
| Amyloid A | 179.5 mg/L (<10.0) |
| Glomerular filtration rate (GFR) | 61.15 ml/min (90-120) |

**Table S2 Pathogen examination in serum on August 5, 2021**

| **Tests** | **Results** |
| --- | --- |
| Toxoplasma IgG antibody | Negative |
| Toxoplasma IgM antibody | Negative |
| Rubella virus IgG antibody | Positive |
| Rubella virus IgM antibody | Negative |
| Cytomegalovirus IgG antibody | Positive |
| Cytomegalovirus IgM antibody | Negative |
| Herpes simplex type I IgG antibody | Positive |
| Herpes simplex type II IgG antibody | Negative |

Abbreviation: IgG, immunoglobulin G; IgM, immunoglobulin M.

**Table S3 Detection of autoantibodies in serum on August 5, 2021**

| **Tests** | **Results (normal values in parentheses)** |
| --- | --- |
| Anti-nuclear antibody | Negative |
| Anti-RNP A antibody | Negative |
| Anti-RNP 68 antibody | Negative |
| Anti-Sm/RNP antibody | Negative |
| Anti-Sm antibody | Negative |
| Anti-SS-A antibody | Negative |
| Anti-Ro-52 antibody | Negative |
| Anti-SS-B antibody | Negative |
| Anti-Scl70 antibody | Negative |
| Anti-JO-1 antibody | Negative |
| Anti-CENP-B antibody | Negative |
| Anti-dsDNA antibody | Negative |
| Anti-chromatin antibody | Negative |
| Anti-ribosomal P protein antibody | Negative |
| IgE | 66.70 IU/ml (1.00-190.00) |
| IgG | 15.40 g/L (7.51-15.60) |
| IgA | 4.17 g/L (0.82-4.53) |
| IgM | 0.920 g/L (0.460-3.040) |
| Complement C3 | 0.888 g/L (0.790-1.520) |
| Complement C4 | 0.302 g/L (0.160-0.380) |
| C-reactive protein | 22.80 mg/L (<8.00) |

Abbreviation: RNP, nuclear ribonucleoprotein; Anti-Sm antibody, Anti-Smith Antibody; Anti-SS-A antibody, Anti-Sjogren syndrome A antibody; CENP-B, Centromere Protein B; dsDNA, double stranded DNA; IgG, immunoglobulin G; IgM, immunoglobulin M.

**Table S4 Detection of cytokines in plasma on August 5, 2021**

| **Tests** | **Results** |
| --- | --- |
| IL-2 | 1.97 pg/ml (0.00-5.71) |
| IL-4 | 1.30 pg/ml (0.00-3.00) |
| IL-6 | 8.51 pg/ml (0.00-5.30) |
| IL-10 | 3.52 pg/ml (0.00-4.91) |
| TNF-α | 1.01 pg/ml (0.00-4.60) |
| IFN-γ | 1.61 pg/ml (0.00-7.42) |

Abbreviation: IL, Interleukin; TNF-α, Tumor Necrosis Factor Alpha; IFN-γ, Interferon Gamma.

**Table S5 HLA-B27 testing with whole blood on August 6,2021**

| **Tests** | **Result** |
| --- | --- |
| HLA-B27 testing | Negative |

Abbreviation: HLA-B27, Human leukocyte antigen B27.

**Table S6 Analysis of tumor markers in serum**

| **Tests** | **Results (normal values in parentheses)** | |
| --- | --- | --- |
|  | June 1, 2021 | June 29, 2021 |
| CEA | 49.97 μg/L (<5.0) | 17.65 μg/L |
| CA19-9 | 19.0 U/ml (<37.0) | 28.6 U/ml |
| CA125 | 479.0 U/ml (<35.0) | 85.4 U/ml |
| CA15-3 | 16.6 U/ml (<31.3) | 18.5 U/ml |
| SCC | 12.6 ng/ml (<1.5) | 1.0 ng/ml |
| CYFRA21-1 | 81.82 ng/ml (<2.50) | 4.30 ng/ml |

Abbreviation: CEA, Carcinoembryonic Antigen; CA19-9, Carbohydrate antigen 19-9; CA125, Carbohydrate antigen 125; CA15-3, Carbohydrate antigen 153; SCC, Squamous cell carcinoma antigen; CYFRA21-1, Cytokeratin-19-fragment CYFRA21-1.
